# Increasing the phylogenetic coverage for understanding broad-scale diversity gradients

**DOI:** 10.1007/s00442-020-04615-x

**Published:** 2020-02-12

**Authors:** Marcell K. Peters, Alice Classen, Jörg Müller, Ingolf Steffan-Dewenter

**Affiliations:** 1 Department of Animal Ecology and Tropical Biology, Biocenter, University of Würzburg, Am Hubland, 97074 Würzburg, Germany; 2 Bavarian Forest National Park, Freyunger Str. 2, 94481 Grafenau, Germany

**Keywords:** Elevational diversity, DNA metabarcoding, Negative density dependence, Productivity hypothesis, Species energy theory, Temperature-speciation hypothesis

## Abstract

Despite decades of scientific effort, there is still no consensus on the determinants of broad-scale gradients of animal diversity. We argue that general drivers of diversity are unlikely to be found among the narrowly defined taxa which are typically analyzed in studies of broad-scale diversity gradients because ecological niches evolve largely conservatively. This causes constraints in the use of available niche space leading to systematic differences in diversity gradients among taxa. We instead advocate studies of phylogenetically diverse animal communities along broad environmental gradients. Such multi-taxa communities are less constrained in resource use and diversification and may be better targets for testing major classical hypotheses on diversity gradients. Besides increasing the spatial scale in analyses, expanding the phylogenetic coverage may be a second way to achieve higher levels of generality in studies of broad-scale diversity gradients.

## Introduction

Since times of Darwin and Humboldt, naturalists are fascinated by the change in the diversity of life when moving from the poles to the equator or from the lowlands to the tops of mountains (Lomolino 2006; Merckx et al. 2015; Colwell et al. 2016). As old as the recognition of the global variation in diversity is the search for a general model to explain it (Brown 2014). A large number of deterministic hypotheses have been suggested from which hypotheses relating diversity gradients to the variation in temperature, primary productivity and biotic interactions are most prominent in the ecological literature (Mittelbach et al. 2007; McCain and Grytnes 2010; Jetz and Fine 2012; Belmaker and Jetz 2015; Fine 2015). The ‘temperature-speciation hypothesis’ relates higher species richness to higher rates of evolutionary diversification (Brown 2014), while the ‘productivity hypothesis’ posits that a higher availability of resources at the base of food webs favors population persistence and species coexistence (Hurlbert and Stegen 2014). The ‘biotic interaction hypothesis’ assumes that increased rates of biotic interactions, like density-dependent mortality induced by antagonists (‘Janzen-Connell Effect’), foster the coexistence of species (Mittelbach et al. 2007). Hundreds of studies conducted along elevational or latitudinal gradients found conflicting support for these and other hypotheses suggesting that there is no single but a bunch of factors that structure levels of biodiversity on earth (Lomolino 2006; McCain and Grytnes 2010; Peters et al. 2016).

While these conflicts can partly be explained by differences in the spatial extend of gradients, differences in sampling completeness, geographic location or biogeographic history of study systems (Rahbek 2005; Nogués-Bravo et al. 2008), much of the limited consensus can probably be attributed to differences in the response of different taxa to environmental drivers (McCain and Grytnes 2010; Peters et al. 2016). However, as most ecological studies are restricted to rather narrowly defined clades (Seibold et al. 2018), e.g., birds or ants, cross-taxon comparisons along the same gradients are scarce. This restriction has obvious practical reasons, such as constraints in funding of field studies, limited taxonomic expertise, or because global data sets on species distributions for invertebrates are very rare.

**Fig. 1:**
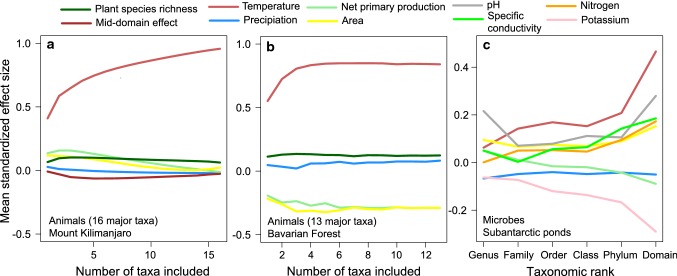
Case studies revealing the dependency of the support for predictors of diversity on the phylogenetic coverage: animal data from an elevation gradient (870–4550 m asl) on Mt. Kilimanjaro (Peters et al. 2016) (**a**), animal data from an elevation gradient (297–1368 m asl) in the Bavarian Forest, Germany (**b**; unpublished data), and, for comparison, for an elevation gradient (10–1038 m asl) on microbes from the subantarctic ponds in Finland and Norway (Yeh et al. 2019) (**b**). In all data sets, mean standardized effect sizes for predictors of species richness were calculated for data sets of increasing phylogenetic coverage, measured either as the number of taxonomic groups included in analyses (**a**, **b**) or by taxonomic ranks (**c**). In **a** and **b,** one up to 16 taxonomic groups like ants, moths, and bats were repeatedly, randomly selected and the total number of species of the pooled assemblages was calculated. The species richness was then analyzed using a multi-model averaging approach in which different additive predictor variables of species richness were used. The mean standardized effect sizes for each predictor variable was then calculated by averaging standardized effect size values across all-taxa combinations for each number of taxa included. In **c**, mean standardized effect sizes were averaged across all taxa for each taxonomic rank. In all three studies, best predictors of species richness strongly differed among single taxa, but with rising phylogenetic coverage, temperature received increasing support. Please note that we here based our approach on taxonomic groups of different ranks as multi-taxa diversity data sets linked to phylogenetic trees are currently not available. While the taxon-focused approach works, the analyses of different, time-defined clades of phylogenetic trees of animal biodiversity would be the better option, as they reflect phylogenetic coverage and relatedness more objectively than taxonomic groups

Here, we sum up conceptual and theoretical arguments exemplifying how the restriction of studies to single, phylogenetically narrowly defined clades constrains assessments of the importance of the drivers of biodiversity gradients: First, due to phylogenetic autocorrelation of niche axes, the influence of drivers of diversity may depend on the phylogenetic scale of analyses, and major classical hypothesis to explain diversity gradients may better fit to phylogenetically broader communities. Second, models explaining patterns for phylogenetically broader communities have a higher level of generality as they predict patterns for larger partitions of the animal tree of life. Building up on evidence from recent studies on plants, animals and microbes (Peters et al. 2016, 2019; Weiser et al. 2018; Yeh et al. 2019) (Fig. 1), we propose studies of multi-taxa animal communities as an alternative to traditional single taxa approaches and as an important step towards a general understanding of the drivers of biodiversity. This approach extends the theory behind biogeographic hypotheses and suggests a new way to perceive universality in the effect of an environmental variable on patterns of biodiversity: while past studies focused on congruence across different taxonomic groups, a driver may be seen as universal if the total diversity of species coexisting in the same area or region is determined by this variable.

## Towards general rules in models of diversity gradients

Even though clade-specific patterns are of high interest and relevance (e.g., ‘what determines species diversity of birds’), a major goal of ecology is to find general rules (McGill 2010). By scaling up from local to regional or global scales, macroecology has strongly expanded our perception about the drivers of diversity at the global level (Kreft and Jetz 2007; Tittensor et al. 2010; Belmaker and Jetz 2015), which may differ to those found at the local level (Brown 1995). While local studies may be of high relevance to understand and predict the distribution of diversity at smaller spatial scales (e.g., national territories), models of global diversity gradients are apparently more general in a sense that they explain patterns across larger regions of the world, encompassing larger environmental gradients (Gaston and Blackburn 2000). These models are not only relevant for basic ecological questions but are of particularly importance in the context of global environmental change (Sundqvist et al. 2013; Mannion et al. 2014). Similar to an increase of the spatial scale, an expansion of the phylogenetic coverage from single taxa to the community level will yield models that apply to communities, which are made up of hundreds or thousands of coexisting and interacting species. As is true for spatial upscaling, these models may not be conforming to models found for narrowly defined clades. Nevertheless, they can be regarded as being more general as they are not restricted to an arbitrarily chosen phylogenetic level but instead predict trends of species diversity across the animal phylogenetic tree of life.

## Why hypotheses on broad-scale diversity gradients often fail to explain species richness

Over the last two centuries, several hypotheses have been proposed to explain the global heterogeneity in biodiversity. Among the hypotheses generally receiving the highest interest are the productivity hypothesis, the temperature-speciation hypothesis, and the biotic interaction hypothesis. These hypotheses have typically been tested in rather narrowly defined and small subsets of the overall phylogenetic diversity of coexisting species. In the next paragraphs, we will elucidate why this restriction conflicts with assumptions of major hypotheses on broad-scale diversity gradients and explain why conservative niche evolution may produce low fits of diversity–driver relationships in phylogenetically narrowly defined clades. While the mechanisms generating the biodiversity patterns vary among the discussed hypotheses, the ultimate reason for the taxon specificity of their support remains the same: conservative niche evolution causing phylogenetic signals in the ecological niches of species (Box 1) (Peters et al. 2016).

### Primary productivity as a driver of species richness

Primary productivity is the total production of autotroph organisms over a defined area and interval of time. The productivity hypothesis, developed by Hutchinson (1959), Wright (1983), Hurlbert and Stegen (2014) and others, posits that areas with high primary productivity can support more species with larger populations than areas of low primary productivity. As the extinction probability of a species in an area is proximately a function of population size, areas of high primary productivity are assumed to support larger numbers of species with viable populations than areas of low primary productivity over time (Kaspari et al. 2000; Hurlbert and Stegen 2014). Likewise, high primary productivity may increase the probability of successful range expansion (Allen et al. 2007), which is a critical step in the diversification process (Price et al. 2014).

The hypothesis that the diversity of a clade is ultimately determined by primary productivity has a central assumption: Either the members of the clade consume the total primary productivity or the fraction of primary productivity consumed by the members of the clade remains approximately constant along the productivity gradient (Hurlbert and Stegen 2014). For phylogenetically narrowly defined clades, both assumptions are unlikely to be met. While the first argument is obviously not valid, studies quantifying the proportional consumption of primary productivity along extensive environmental gradient are absent. However, there is a multitude of empirical data and established knowledge indicating strong shifts in the relative amount of resources consumed by a narrowly defined clade along broad environmental: e.g., bees are dominating consumers of pollen and nectar in warm climates, but syrphid flies and other Diptera are of increasing importance in cold climates (Kühsel and Blüthgen 2015; Rader et al. 2016). The true dung beetles and termites are dominating feeders of dung and dead plant matter in hot ecosystems, like tropical savannah or lowland tropical rainforests but they are absent or scarce in higher latitudes and high elevations where, for instance, coprophagic flies and Collembola proliferate (Palin et al. 2011; Farwig et al. 2014; Röder et al. 2017). While the proportional consumption of a closely defined resource like flower nectar/pollen, dung or dead plant matter already strongly changes along climatic gradients, the variation in the proportional consumption of the total primary productivity by a narrowly defined clade can be assumed to be even higher.

**Fig. 2:**
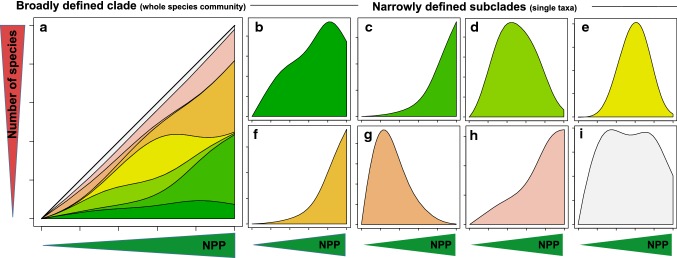
The importance of net primary productivity as a driver of diversity may depend on the phylogenetic coverage of animal communities. Narrowly defined clades use only small fractions of the food resources available in ecosystems. Moreover, this fraction may vary along broad climatic gradients if climatic niches evolve conservatively. In consequence, even if net primary productivity (NPP) ultimately limits the species richness of a phylogenetically broadly defined clade (**a**), this may not be evident at the level of more narrowly defined subclades (**b**–**i**). Panel (**a**) shows the cumulative species richness of a whole animal community (phylogenetically broadly defined clade) against NPP, with single subclades represented by different colors. Panels (**b**–**i**) present NPP–richness relationships of individual subclades

The ultimate reason for changes in the proportional use of primary productivity along broad environmental gradients is that species within a narrowly defined clade share similar, ancestral traits and respond similarly to the abiotic or biotic environment. Animals of narrowly defined clades effectively consume resources not over the full but over a partition of a climatic gradient. Moreover, species within narrowly defined clades often share similar food niches such that these clades only consume a tiny and probably variable partition of the total primary productivity. For example, despite their Mesozoic origin, nearly all extent species of leaf beetles are angiosperm herbivores. Broadening the definition of a clade leads to an extension of total niche space over the full productivity gradient and an increase in the clades’ proportional consumption of primary productivity (Hurlbert and Stegen 2014). Therefore, if primary productivity (or the amount of resources) determines how many species can coexist in an area, it is more likely to find high correlation coefficients for phylogenetically broadly defined clades than for narrowly defined clades (Fig. 2). In other words, primary productivity should better predict the diversity of all Lepidoptera than the diversity of a given butterfly species; and better the diversity of all consumers than the diversity of Lepidoptera.

### Temperature as a driver of species richness: temperature-speciation hypothesis

**Fig. 3:**
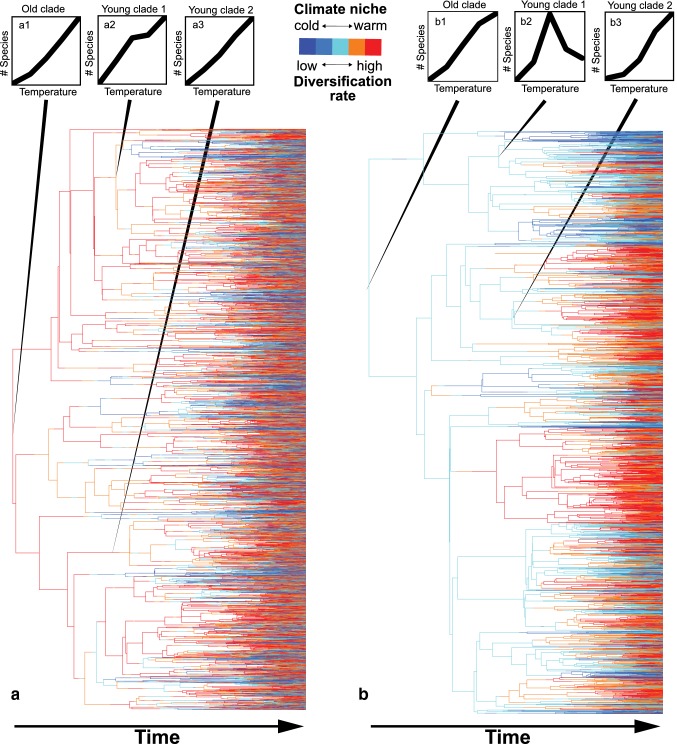
Phylogenetic coverage and its effects on diversity–temperature relationships under progressive (**a**) or conservative trait evolution (**b**). Shown are random *tree.musse* simulations (R package diversitree) of pure temperature-dependent diversification with the following parameters: speciation rate (*λ*) 0.18–0.26 from cold (blue) to warm regions (red), extinction rate in all regions = 0.1 and progressive, gradual evolution (left; rate of lineage niche transition = 0.26) or conservative, gradual evolution (right; rate of lineage niche transition = 0.04 for adjacent temperatures). The simulations ran until 8000 extend lineages were generated. **a** When niche evolution is progressive (climate niche transition rate ≈ diversification rate) the temperature–diversity relationship can manifest quite rapidly such that it is not only evident in the most inclusive clade (**a1**) but also in younger subclades (**a2**, **a3**). **b** However, when niche evolution is conservative (climate niche transition rate  << diversification rate), the effect of temperature on diversity is evident in the phylogenetically broadest clade (**b1**) but not necessarily in more narrowly defined subclades of younger age (**b2**, **b3**). Please note that the diversity patterns in all subpanels are produced by the same diversification model (*λ* ~ *F*(temperature)) and not by any other process

The temperature-speciation hypothesis states that higher mutation rates and shorter generation times in warm climates lead to faster genetic divergence among populations and higher rates of speciation (Allen et al. 2006; Brown 2014; Classen et al. 2015). The temperature-speciation hypothesis, thus, predicts a monotonous increase of species richness with increasing temperature along climatic gradients. But will temperature-related speciation rates always lead to monotonous increases of species richness with temperature? A simple model in which the environmental temperature determines rates of linage diversification and in which only the rate of transition of environmental niches (the probability to change the temperature niche) is adjusted shows that the finding of positive diversity–temperature relationship is largely depending on the interplay of clade age (time for diversification) and the rate at which lineages evolve environmental niches in relation to the rate of diversification (Fig. 3). If rates of niche transition are relatively high in comparison to diversification rates, a positive richness–temperature relationship can manifest quite rapidly such that positive richness–temperature relationships are not only found for broadly defined clades but also for phylogenetically narrow subclades of young age (Fig. 3a). However, if rates of niche transition are relatively low in comparison to diversification rates, i.e., when simulating some moderate degree of niche conservatism, hump-shaped or even negative temperature–diversity relationships are frequently found in subclades of younger age, in particular in clades whose ancestral niche is situated in cold environments (Fig. 3b). Recent analyses suggest that conservative niche evolution is the rule rather than the exception in the evolution of life (Wiens et al. 2010). For example, speciation in plants is mostly associated with biome stasis and scarcely with a biome shift (Crisp et al. 2009). In amphibians, realized climatic niches show a significant phylogenetic signal (Hof et al. 2010). In north American salamanders, the specialization of species to environmental conditions at mid-elevational montane regions is suggested to have limited their dispersal to other elevational zones (Kozak and Wiens 2010). Conservatism in this climatic adaptation over time leads to a greater buildup of species in the ancestral environment of salamanders (Kozak and Wiens 2010). Even though temperature increases speciation rates, speciation and the survival of new species may largely be constrained to the ancestral ecological niche space of a clade which only slowly expands over time. In consequence, diversity will only be strongly positively correlated with temperature in clades which cover areas of large temperature variation for longer periods of evolutionary time. This is necessarily more likely the case for phylogenetically broadly than for narrowly defined clades. Therefore, if temperature is the main driver of animal diversity, the chance to detect positive temperature–diversity relationships is higher for phylogenetically broadly than for narrowly defined clades.

### Biotic interactions/negative density dependence as a driver of species richness

**Fig. 4:**
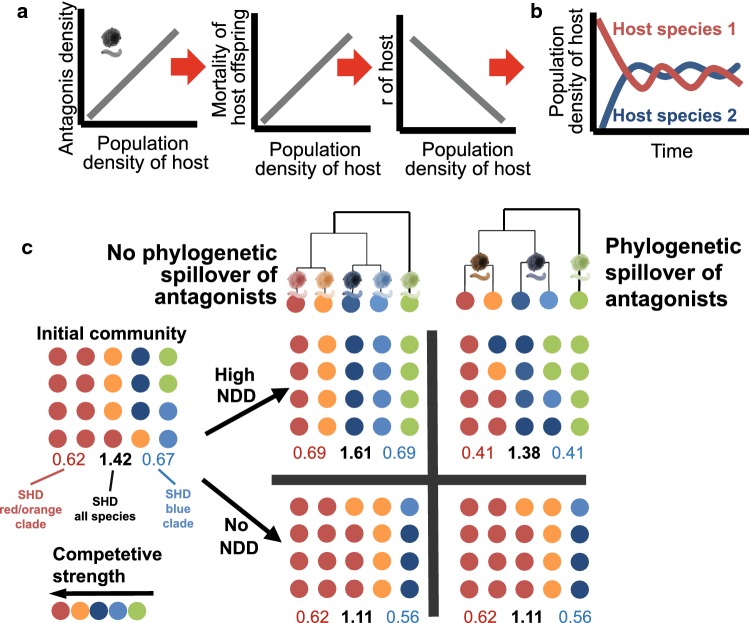
Negative density dependence as a driver of diversity and the importance of the phylogenetic relatedness. **a** Negative density dependence (NDD) is a mechanism by which a high local abundance of a host/prey (for reasons of simplicity only ‘host’ is shown in the figure) leads to high mortality of its offspring and consequently to lowered rates of population growth (*r*). Higher mortality of hosts/prey is assumed to be caused by increased predation pressure of antagonists on abundant host/prey species. **b** This mechanism prevents population densities of best competitors to permanently reach high levels (host species 1 in **b**), as well as the competitive exclusion of other host/prey species (host species 2 in **b**) which maintains high levels of species diversity over time. **c**, left, Traditionally, the spillover of antagonists among related host/prey species is not considered for predicting effects of negative density dependence. Under this traditional model of species-specific antagonist activity, diversity gradients caused by NDD can manifest both at the level of the complete clade and subclades. **c**, right, However, a magnitude of studies suggest that most antagonists are not species specific but attack multiple related host/prey species. Under these circumstances, NDD may lead to reduced population growth of all species sharing antagonists (here it is assumed that the two species in the blue and the two species in the red/orange clade share antagonists). In consequence, the effect of negative dependence on species diversity may only be detectable when calculating diversity estimates of all species in a community (black numbers) but not within single subclades (red, blue, green). Species slightly vary in the degree of competitiveness (red > orange > dark blue > bright blue > green) causing increased dominance and consequently loss of species diversity in the absence of NDD. Each dot represents an individual within a local community of 4 × 5 individuals. Individuals are derived from one of five host or prey species from three subclades (blue, red, green). Numbers below each community represent diversity estimates (Shannon–Wiener diversity index, SHD)

A third, major concept to explain the increase of diversity from temperate to tropical regions is the increase in rates of species interactions, particularly the increase in rates of those interactions that maintain high levels of diversity (Mittelbach et al. 2007; Schemske et al. 2009). The probably most important mechanism in this respect is negative density dependence, whereby abundant species have lower reproduction rates due to increased competition or higher attack by host-specific antagonists [also known as the Janzen–Connell hypothesis] (Bagchi et al. 2014; Liu et al. 2015). Variation in the extend of negative density dependence is assumed to be a key determinant of the geographic variation in local plant species richness (Johnson et al. 2012; LaManna et al. 2017) and has been hypothesized to be related to temperatures’ general positive effect on metabolism, activity and population growth of ectothermic organisms (Brown 2014). While past studies mostly considered conspecific negative density dependence (Johnson et al. 2012), a potentially highly relevant but little understood aspect of negative density dependence is the degree to which it is affected by the relatedness of coexisting species (Fig. 4). As phylogenetically closely related species share similar traits and environmental distributions, they compete, on average, more intensively for space or resources and share more antagonists than less related species (Gilbert and Webb 2007; Davies and Pedersen 2008; Wiens et al. 2010; Parker et al. 2015). There is an extensive literature emphasizing effects of phylogenetic relatedness on competition (known as the ‘competition-relatedness hypothesis’ and ‘limiting similarity hypothesis’), attack rates by enemies or mammal infection with diseases (Gilbert and Webb 2007; Davies and Pedersen 2008; Parker et al. 2015). For example, parasitoid fly and wasp species attack multiple host species of the same genus or family but scarcely those of different families (Smith et al. 2006, 2008). In mammals, infectious diseases are often shared between species that are closely related (Davies and Pedersen 2008; Makanga et al. 2016). Moreover, even if an antagonist is currently specific to a particular host species, the probability that the antagonist spreads over to a second host is higher among related than among phylogenetically independent taxa (Wiens et al. 2010; Makanga et al. 2016). For example, most clades of malarian blood parasites are restricted to either mammals (or subgroups of mammals or birds), birds or reptiles with scarce events of host switches among these groups (Martinsen et al. 2008). Despite a vast literature strengthening the importance of phylogenetic relatedness for transmission of antagonists, phylogeny has scarcely been implemented in tests of negative density dependence.

Importantly, if the agents causing negative density dependence target multiple related species rather than single species (‘apparent competition’), a restriction of studies on diversity gradients to narrowly defined clades may hinder the detection of negative density dependence as a general driver of species richness (Fig. 4). Due to the spillover of antagonists to related taxa, the costs of high abundance may be restricted not only to an abundant species per se but also to its phylogenetic relatives (Parker et al. 2015; Downey et al. 2018). Phylogenetically distinct species, on the other hand, may profit more strongly from high rates of density-dependent mortality of an abundant species than species from the same genus or family. This has important consequences for the phylogenetic scale at which effects of negative density dependence can be observed: A positive relationship between strength of negative density dependence and species richness may not be observed within a narrowly defined clade of species which share many of their antagonists. Phylogenetically broad communities including distantly related taxa with different antagonist communities may be alternative target levels to identify positive effects of negative density dependence on species diversity.

## Practical issues: the way forward

Due to these theoretical and conceptual reasoning, ecological studies incorporating all animal clades will be able to find new answers concerning the drivers of species richness along elevational or latitudinal gradients. However, all-taxa approaches are difficult to implement due to constraints in funding, time-consuming species identification, and lack of taxonomic expertise for invertebrates in many regions of the world (Didham et al. 2013). However, proximate patterns of community-level species richness can already be derived with only a small fraction of monetary resources required for all-taxa approaches by sampling not all species but a random sample of species from communities and by making use of metabarcoding approaches (Ji et al. 2013).

If communities are sampled using standardized protocols, increasingly large random samples of species from communities will produce increasingly correct estimates of the true community-level diversity distribution (Peters et al. 2016). A critical step will be to sample species randomly in the field and a variety of sampling techniques may be necessary to sample species of major taxonomic groups relatively independent of phylogenetic clade membership and dominant microhabitat use (Basset et al. 2012). Random samples (of a standardized size) of the pooled individuals per study site, which are then identified to the species level, and used to estimate asymptotic species richness (Chao and Chiu 2016) could provide standardized estimates of community-level diversity. This approach will, however, tend to work better for organisms for which body size and quantity in samples are not extremely inhomogeneous. Less efficient as a fully random sampling scheme but more practical is the implementation of stratified random sampling scheme, in which taxa of varying taxonomic rank are selected in an unbiased way (Peters et al. 2016). Both, a random sample and a stratified random sample of animal species may allow a representation of the community-level diversity even when only a partition of all species or taxonomic units have been sampled (Peters et al. 2016). Alternatively, the identification of ‘indicator taxa’, whose species richness is closely related to those of the complete animal community, could also provide a cost-efficient approach to assess community-level diversity. However, correlations between the diversity of single taxa and whole communities would first have to be established. Generally, the methodology (random or stratified samplings, or indicator taxa) to achieve representative estimates of community-level species richness has to be developed and tested for different environmental gradients and biogeographic regions.

Metabarcoding of DNA may be a cost-efficient option to derive estimates of community diversity from samples of collected specimens (Ji et al. 2013; Kress et al. 2015). Sequences obtained by metabarcoding approaches may be processed with existing bioinformatic pipelines to eliminate sequencing errors and to derive molecular operational taxonomic unites most likely representing species (Leray et al. 2013; Leray and Knowlton 2015). However, it has to be considered that metabarcoding often comes along with a loss of biological information. Particularly in the biodiverse tropical ecosystems, where a large percentage of invertebrate species is still unknown or not described, DNA barcodes can often not be linked to described species and species traits. This information, however, can be of large help in understanding the drivers of diversity gradients. Moreover, selectivity of primers and amplification protocols in metabarcoding may produce biases in species sampling which may remain obscure if no quality checks are conducted. Despite its deficits, a consequent application of metabarcoding of species communities may strongly reduce time for sample processing and identification and may allow standardized estimates of community-level diversity along multiple latitudinal or elevational gradients. Metabarcoding may, therefore, reveal results complementing those of more detailed analyses of particular taxonomic groups (Kress et al. 2015).

Metaanalysis allows inferring the support for hypotheses across a large number of different, often independently collected data sets. Its application provided new insights on the generality of broad-scale diversity gradients (e.g., Hillebrand 2004). However, results of meta-analyses will differ from those of true multi-taxa or all-taxa community biodiversity analyses. First, they typically do not incorporate differences in the diversity among taxa, so that results on little diverse taxa or narrowly defined clades contribute the same to parameter estimates than highly diverse taxa or phylogenetically broadly defined clades, respectively. Second, as meta-analyses are calculated with independent data sets on diversity gradients, they are unable to incorporate the influence of interactions among taxonomic groups for the establishment of diversity gradients (as exemplified for the productivity and biotic interaction hypotheses). The difference between results of multi-taxa approaches and meta-analyses becomes evident in Fig. 1: values on the far left side of the *x* axis show standardized parameter estimates for potential diversity drivers averaged across single taxa (i.e., results that would reflect those from meta-analyses). In contrast, the values on the far right side of the *x* axis show parameter estimates of true multi-taxa approaches in which diversity values for all taxa within a community are summed up before parameter estimates were calculated. Please note that parameter estimates found for some predictor variables (here temperature) are much higher in multi-taxa approaches than in meta-analyses of data sets, while the support values for other predictor variables decrease.

## Conclusion

The last decade has seen an increasing interest in the search for general drivers of biodiversity but past studies on single taxa revealed little congruency in support for individual hypotheses. Undoubtedly, understanding the mechanisms shaping local, single clade-specific patterns (e.g. ‘what determines species diversity of birds in central Europe?’) is of high interest and relevance in ecology and conservation biology, as models effectively improve the predictability of how specific clades and the functions they are providing in ecosystems are affected by environmental changes. However, assessing the impact of global change on entire ecosystems and ecosystem functions requires a better understanding of the general drivers of species richness across spatial and phylogenetic scales. We argue here that adopting a multi-taxa community-level perspective may contribute to the development of more general rules in the studies of diversity gradients. Based on theoretical arguments, a community-level perspective may not lead to a counterbalancing of different drivers but may increase the statistical support for a single or few drivers of species richness which shaped large parts of the temporal and spatial evolution of animal diversity on earth. While we exemplified the potential importance of phylogenetic coverage for tests of three major biogeographic hypotheses, the principal ideas described herein may also be transferred to other hypotheses to explain diversity gradients (e.g., area hypothesis, plant diversity hypothesis). Moreover, the concept which we mainly described here for animals may also be applied to other kingdoms in the tree of life, as plants, fungi or Eubacteria. While for these groups much more multi-taxa data sets are currently already available, the influence of phylogenetic scale on the inference on drivers of species diversity has only recently been in the focus of analyses (Weiser et al. 2018; Yeh et al. 2019).

Even though further studies are necessary to understand the extend and strength of conservative niche evolution across the phylogenetic tree of life, available data and theory suggest that high phylogenetic relatedness sets strong constraints on resource use and evolution of niche space which necessarily leads to incongruence in the drivers of species richness among individual clades. Increasing the phylogenetic coverage in ecological studies, for example, by a global assessment of multi-taxa communities on all continents and in all climate zones using standardized methods could provide new perspectives on the drivers of broad-scale diversity gradients.

**Fig. 5:**
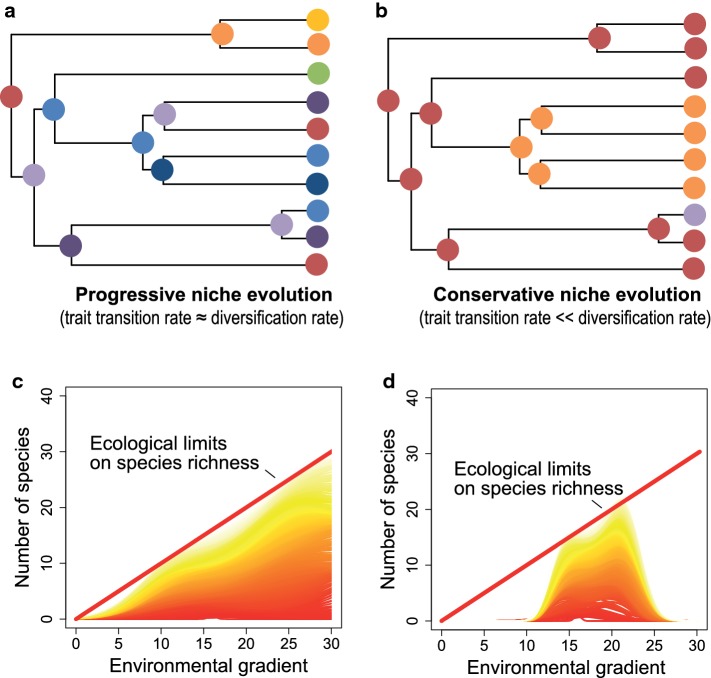
Consequences of conservative and progressive niche evolution for shaping species richness gradients along environmental gradients. It is assumed that environmental conditions constrain the number of coexisting species along the environmental gradient (bold red line). If environmental niches evolve fast relative to rates of diversification (progressive niche evolution), species can distribute rapidly over the full environmental gradient and reach local species limits (**a**). A consequence is a high fit of species richness to the limits set by the environment (**c**). However, this assumption may in reality not often been met as conservative niche evolution is the rule rather than the exception in the diversification of life. If environmental niches of species evolve slowly relative to rates of diversification (conservative niche evolution), a taxon may not reach ecological limits on species richness in all parts of the environmental gradient (**b**). This may lead to a low fit of species richness to the species limits set by the environment (**d**). Curves in heat color show the progress of species richness patterns over time; red = old, yellow = recent)

Box 1: Phylogenetic autocorrelation of ecological niches An increasing number of studies relating ecological data to phylogenies reveal that niches of species and related traits evolve largely conservatively (Wiens et al. 2010). Even though examples, in particular from isolated islands, indicate that rapid evolution of niches is principally possible (Fig. 5b), evidence is mounting that key niche axes, like the food niche or climatic niches, remain mostly consistent or only change slowly over several millions of years of diversification (Fig. 5a). As Wiens and colleagues underscored: ‘niche conservatism in a species or clade may be most apparent when contrasted with an alternative set of ecological conditions or resources that they fail to occupy or utilize, and which are instead occupied by other species or clades’ (Wiens et al. 2010). For example, when comparing the niche spaces used by two randomly selected butterflies to those of a butterfly and a mammal species it becomes obvious that even though evolution may have had millions of years for diversification, food or climatic niches within narrowly defined clades remain relatively similar. But why? Most important, the traits defining the ecological niches are inherited, and mostly evolve gradually and slowly in relationship to the rates of diversification. Several studies revealed that niches remain even more similar than predicted by a Brownian (random) model of trait evolution, a process or pattern which some consider as niche conservatism in a strict sense (Losos 2008). The ultimate reasons for this type of niche conservatisms include limitations in genetic variation, natural selection against extreme phenotypes, dominant gene flow from the center of populations, tradeoffs between drivers of trait evolution and others (Wiens and Graham 2005; Wiens et al. 2010; Crisp and Cook 2012). Niche conservatism can have considerable consequences for studying the importance of the environmental drivers of biodiversity, for example narrowly defined clades of related species (1) only use a partition of the food resources which are principally available in ecosystems, (2) are only distributed over a partition of the climatic gradients principally available along latitudinal or elevational gradients, or, (3) share similar enemy predators, parasitoids or pathogenic microbiota.
